# RAQ–A Random Forest Approach for Predicting Air Quality in Urban Sensing Systems

**DOI:** 10.3390/s16010086

**Published:** 2016-01-11

**Authors:** Ruiyun Yu, Yu Yang, Leyou Yang, Guangjie Han, Oguti Ann Move

**Affiliations:** 1 Software College, Northeastern University, Shenyang 110819, China; yangleyou@163.com (L.Y.); annmove@swc.neu.edu.cn (O.A.M.); 2 Department of Computer Science, Rutgers University, New Brunswick, NJ 08854, USA; yangyu.9415@rutgers.edu; 3 Department of Internet of Things Engineering, Hohai University, Changzhou 213022, China; hanguangjie@gmail.com

**Keywords:** air quality prediction, random forest, point of interest, traffic

## Abstract

Air quality information such as the concentration of PM_2.5_ is of great significance for human health and city management. It affects the way of traveling, urban planning, government policies and so on. However, in major cities there is typically only a limited number of air quality monitoring stations. In the meantime, air quality varies in the urban areas and there can be large differences, even between closely neighboring regions. In this paper, a random forest approach for predicting air quality (RAQ) is proposed for urban sensing systems. The data generated by urban sensing includes meteorology data, road information, real-time traffic status and point of interest (POI) distribution. The random forest algorithm is exploited for data training and prediction. The performance of RAQ is evaluated with real city data. Compared with three other algorithms, this approach achieves better prediction precision. Exciting results are observed from the experiments that the air quality can be inferred with amazingly high accuracy from the data which are obtained from urban sensing.

## 1. Introduction

**Figure 1:**
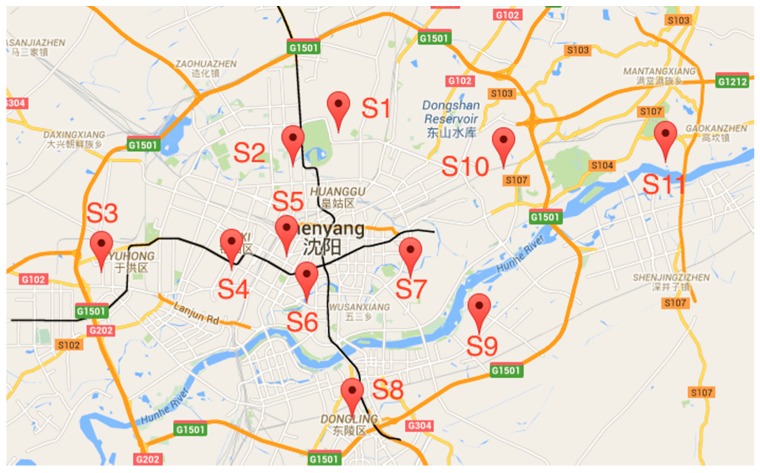
Monitoring station locations in Shenyang city (China).

As urbanization leads to urban community growth, the transportation infrastructure dependent on fossil fuels also expands consequently [1]. The popularity in vehicle use gives rise to an increase in traffic related pollutant emissions. Urban air pollution is a major problem in both developed and developing countries, as atmospheric pollutants have a great effect on human health. Numerous illnesses such as lung cancer may be caused by various atmospheric pollutants [2]. In addition, some other serious environmental problems can also result from air pollution, such as acid rain and the greenhouse gas effect. For example, SO_2_ and NO_2_ are the main causes of acid rain [3], while CO_2_ and N_2_O are the main reasons for the greenhouse gas effect [3]. Recently, especially in China, environmental problems have become a major concern in big cities such as Beijing and Shanghai, where the primary sources of pollutants include exhaust emissions from Beijing's more than five million motor vehicles, coal burning in neighboring regions, dust storms from the north and local construction dust [4]. A particularly severe smog engulfed the Beijing for weeks in early 2013, elevating public awareness to unprecedented levels and prompting the government to roll out emergency measures [4]. Air pollution monitoring is thus becoming more and more significant. Real-time air quality information, such as the concentration of PM_2.5_, PM_10_ and NO_2_, is an important aspect for pollution management and protecting human beings from damages caused by air pollutants. Considering the significance of air quality, governments take measures to monitor it through establishing air quality monitoring stations. However, because of the high expense to start up and maintain these facilities, there are not sufficient stations in cities. For example, Figure 1 shows the Google Map of Shenyang City. The red pins represent the 11 air quality monitoring stations. Among them, S1 is located in a college; S2, S3, S4, S6, S8 are located on the roofs of buildings; S5, S9 are located along roads; S10 is located in a park; S11 is located near factories. These only 11 stations that cover more than three thousand square kilometers of downtown area in Shenyang. Another example is to compare London and Beijing. The area of Beijing is 10 times bigger than London but the number of monitoring stations is less than one fourth of London’s [5]. One station can only monitor an area of limited size, therefore precise air quality reports for many areas cannot be generated.

**Figure 2:**
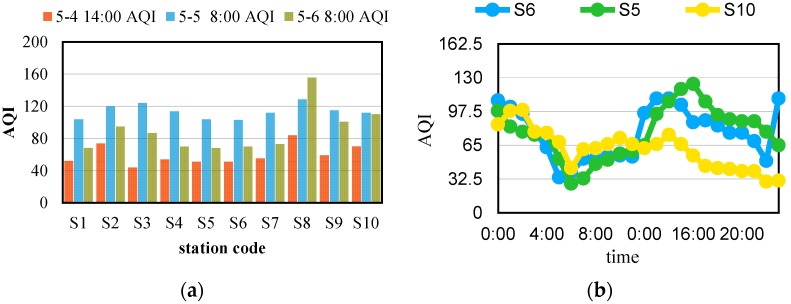
(**a**) AQI Samples in Shenyang; (**b**) AQI Trend on 12 May 2015 in Shenyang.

Figure 2a shows samples of the AQI data of 10 stations in different locations. The x-axis denotes the different stations and the y-axis denotes AQI. Three bars in colors denote the AQI at different times. As demonstrated in Figure 2a, stations at different locations can differ a lot at the same time such as S7 and S8 on 6 May 2015 [6]. Air quality on continuous two days can also display big jumps such as AQI at S3 which raised from 55 to 408 in the morning between 6 May 2015 and 7 May 2015 [6]. Figure 2b shows the ways in which air quality changes follow different rules in different locations. For example, no matter whether stations are a short distance apart like S5 and S6 or a long distance like S5 and S10, they showed different changes between points in time.

It is hard to reflect these changes in a general function which can be applied to all the locations, therefore, we cannot come up with a general formula to predict the air quality in a certain time slot. Therefore, how to infer the air quality in the blank areas is a challenging and meaningful topic. In this paper, we come up with an algorithm to infer the air quality indications throughout the city. In an urban sensing system, an algorithm (RAQ) based on a random forest concept is proposed to predict the urban area air quality through the use of historical air quality data, meteorology data, historical traffic and road status as well as POI distribution information. These data are collected from all kinds of urban sensors such as weather monitoring stations. This method hides all these kinds of inaccessible factors in the traditional mathematic models. In practical applications, we cannot take all the factors such as vehicle emissions and factory emissions into count, as it is hard to get accurate data about these factors. This kind of replacement is not only good for the computation but also good for increased prediction accuracy. At the same time, all the features used in this paper are much cheaper than the accurate measured data from monitoring stations. No equipment cost is required in this approach. As for the accuracy, this algorithm performs better than some other classical ones and the overall results can provide meaningful references to citizens. Regarding the scalability and expansibility, more possible related features such as human mobility can be input into this algorithm without significant changes. The algorithm itself is also robust enough for even higher dimensions.

The remainder of this paper is organized as follow: Section 2 presents related work. The problem description and formulation are presented in Section 3. In Section 4, the system framework and the RAQ algorithm is proposed. Extensive experiments are implemented in Section 5. We conclude and outline the directions for future work in Section 6.

## 2. Related Work

In the past decades, many studies on air quality inference have been done using approaches such as dispersion models, satellite remote sensing and wireless sensor networks. Air pollution dispersion models are tools that use a mathematical model such as the Box model [7], Gaussian model [8], Lagrangian model [9], Eularian model [10], SLAB model [11] or some mixed models. to simulate how air pollution disperses in the atmosphere. The classical dispersion models are mainly functions of meteorology, traffic volumes, building distributions and so on. These models depend mainly on experience and the parameters above to simulate the pollution dispersion, but some other potential factors are not taken into consideration such as human mobility and concentrations. In the meantime, dispersion models depend on access to relatively accurate data, such as the strength of pollutant sources, wind speed, traffic emissions and so on, which accuracy cannot be guaranteed in certain conditions. For example, wind speed may vary a lot in different regions because of the obstructions of buildings, and their roles in determining the modified wind circulation between and over structures. Accurate traffic emissions are also hard to obtain. We can only estimate the value according to the fuel consumption and distances travelled.

Satellite remote sensing technology is another possible way to monitor air quality. Research has developed quickly using satellites to monitor air conditions in the past decades. For example, Liu *et al.* came up with an approach using satellite remote sensing technology to test the thickness of PM_2.5_ on the ground [12]. Similarly, Martin *et al.* came up with a way of using satellite remote sensing technology to test some ground air pollutants, including CO, NO, SO_2_ and so on [13]. Pawan *et al.* used this technology to evaluate the air conditions of every city [14]. These methods mainly use satellite remote sensing technology to directly measure the concentration of certain air pollutants by analyzing the images obtained by the satellites to estimate the concentrations of air pollutants. However, many air quality managers are not yet taking full advantage of satellite data for their applications because of the challenges associated with accessing, processing, and properly interpreting observational data. That is, a certain degree of technical skill is required on the part of the data end-user, which is often problematic for organizations with limited resources [15]. 

Sensor networks have also been studied extensively because of their broad applicability and enormous application potential in areas such the environmental monitoring field. A Wireless Sensor Network Air Pollution Monitor System (WAPMS) was deployed on the island of Mauritius for monitoring air quality [16]; distributed infrastructure-based wireless sensor networks and grid computing is also used for monitoring the air quality of London [17]. Rajasegarar *et al.* also used wireless sensor networks to monitor air pollutants [18]. However, sensor networks require a large number of sensor devices, and can only be deployed in a small range, such as indoors and in small areas. For a city and other large areas, if using cheap sensors with single function, we cannot get information about all kinds of air pollutants. If using sensors with complex functions such as monitoring stations, infrastructure construction and maintenance costs make it difficult to promote wireless sensor networks for a wide usage range. It is the same reason which limits the number of stations in cities of China.

Besides all the methods above, participatory sensing is also an important approach for air quality prediction. With the popularity of smart devices, participatory sensing and crowdsourcing has been a hot topic of discussion in recent years. People see unlimited possibilities in smart devices. A personalized mobile sensing system (MAQS) was proposed for indoor air quality monitoring [19]; a system based on smart phones and monitoring sensors has also been used to monitor outdoor air quality [20]; noise pollution is also monitored using mobile phones [21]. Sivaraman *et al.* used a participatory sensor system to monitor air pollutants in Sydney (Australia) [22]. However, most current smartphones does not carry air pollutant sensors, so the sensing devices required for the system need external sensing modules which leads to extra costs. Besides the high expense, user participation and the accuracy of the data are problems that remain to be solved.

Recently, urban computing has been one of the ways to solve problems in cities. Yuan Jing *et al.* proposed an algorithm to infer the functional areas of cities by using trajectories [23]; Zheng *et al.* made use of the city daily data to infer urban air quality [24,25]. However, similarly, urban computing also requires pre-installed urban sensors such as GPS devices. For instance, when inferring the air quality, Zheng made use of months of data collected from the GPS installed in taxis in Beijing. This is an important limitation that prevents the promotion of this approach because in most cities we cannot access the GPS information of taxis. Spatiotemporal data analysis is also an important aspect for air quality prediction. Chen *et al.* established a spatiotemporal data framework named BigSmog to provide China smog analysis [26]. Zhu *et al.* proposed Granger-causality-based air quality estimation with heterogeneous spatiotemporal data [27]. Some other studies [28,29] also analyzed spatiotemporal data to generate air pollutant distributions.

## 3. Problem Description and Definition

### 3.1. Definition

#### 3.1.1. Air Quality Index

An air quality index (AQI) is a number used by government agencies to communicate to the public how polluted the air is currently or how polluted it is forecasted to become [30]. As the AQI increases, an increasingly large percentage of the population is likely to be exposed, and people might experience increasingly severe health effects. Different countries have their own air quality indices, corresponding to different national air quality standards. In this paper, we use the standard of China, where the AQI is based on the levels of six atmospheric gases, namely sulfur dioxide (SO_2_), nitrogen dioxide (NO_2_), suspended particulates smaller than 10 μm in aerodynamic diameter (PM_10_), suspended particulates smaller than 2.5 μm in aerodynamic diameter (PM_2.5_), carbon monoxide (CO), and ozone (O_3_), measured at the monitoring stations throughout each city [31]. The AQI value is calculated per hour according to a formula published by China’s Ministry of Environmental Protection [31]. AQI is the maximum value of $IAQI_{p}$ which is a reference value of one air pollutant p:

(1) $$AQI=max\left\{IAQI_{1},IAQI_{2},IAQI_{3},\ldots ,IAQI_{n}\right\}$$

(2) $$IAQI_{p}=\frac{IAQI_{H_{i}}-IAQI_{L_{o}}}{BP_{H_{i}}-BP_{L_{o}}}\left(C_{p}-BP_{L_{o}}\right)+IAQI_{L_{o}}$$

where $C_{p}$ is mass concentration value of the air pollutant p, $BP_{H_{i}}$ is the high value of the concentration limit which can be checked in the reference table from the paper [31], $BP_{L_{o}}$ is the low value of the concentration limit which can be checked in the reference table from [31], $IAQI_{H_{i}}$ is the corresponding value of $BP_{H_{i}}$ in the same reference table, $IAQI_{L_{o}}$ is also the corresponding value of $BP_{L_{o}}$ in the reference table. Table 1 shows the relationship between AQI values and air pollution levels which are marked by different colors. In this way, air quality prediction can be treated as a classification problem so that we only need to match the air quality index to different classification levels in Table 1. The six levels in Table 1 represent six AQI levels.

**Table 1:** AQI classification.

AQI	Air Pollution Level
0–50	Excellent
51–100	Good
101–150	Lightly Polluted
151–200	Moderately Polluted
201–300	Heavily Polluted
300+	Severely Polluted

#### 3.1.2. Traffic Congestion Status

**Figure 3:**
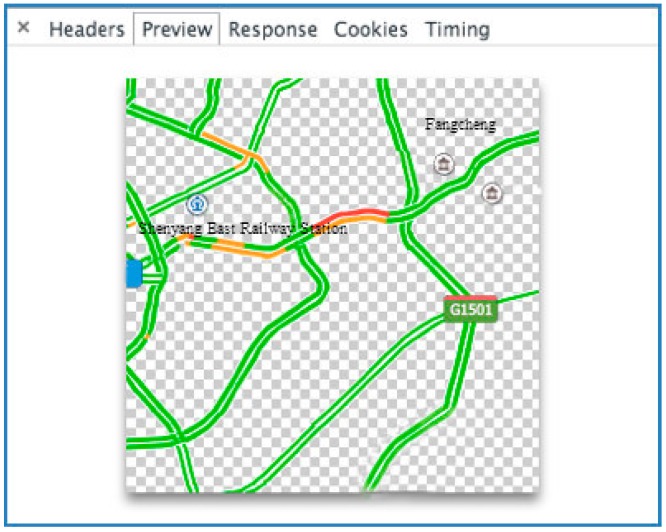
A TCS graph.

Traffic Congestion Status (TCS) describes the traffic conditions on a certain road. Different colors denote different levels of congestion. For example, Figure 3 shows an example of a TCS graph.

#### 3.1.3. Point of Interest

**Figure 4:**
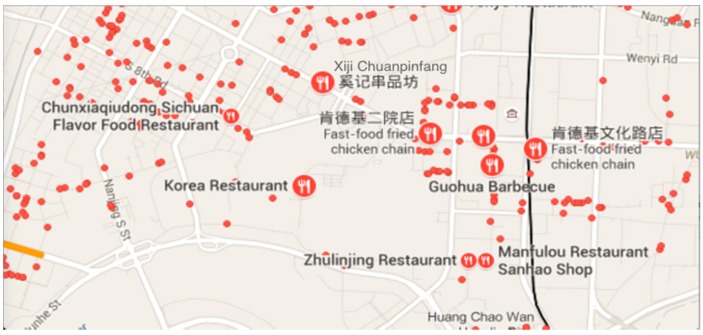
POI near the Sanhao street of Shenyang city.

A point of interest, or POI, is a specific location that someone may be interested in. For example, restaurants and shopping malls surrounding us are POI. Figure 4 presents the restaurant locations around Sanhao Street of Shenyang on Google Maps. 

### 3.2. Problem Formulation

This paper uses urban sensing data to solve the problem of air quality inference which means to infer the unknown air quality of areas by using all kinds of data. These data affect either the sources of air pollution such as traffic emissions and point of interest distribution or their results such as the air quality index, so establishing the relationship between these data and air quality is the key to this kind of approach. The RAQ algorithm collects several kinds of related data including air monitoring station data (AQI), meteorology data (MD), traffic (TCS), road information (RI) and POI data. All these data are fetched at intervals of one hour. We divide the city into grids (G) and each grid is regarded as one unit. Those grids (G_1_) with air quality monitoring stations generate the data with the label AQI while the grids (G_2_) without stations generate the data used for prediction. Data from G_1_ are used for training our learning model and data from G_2_ are input into the model to generate the predication value. The only difference of data from G_1_ and G_2_ is data from G_1_ are labeled as an AQI value. The results are given as different AQI levels. If the actual value from monitoring stations belongs to this AQI level, then we know the prediction is right. Otherwise the prediction is wrong.

This problem can be formulated as follows: given a collection of grids *G* = *G*_1_ ∪ *G*_2_ (|*G*_1_|≪ |*G*_2_|), where *g*_1_*·AQI* (*g*_1_ ∈ *G*_1_) is known and *g*_2_*·AQI* (*g*_2_ ∈ *G*_2_) is unknown, *g·MD*, *g·TCS, g·RI* and *g·POI* are known (*g* ∈ *G*), RAQ aims to predict *g*_2_*·AQI* at intervals of one hour.

## 4. RAQ Algorithm

**Figure 5:**
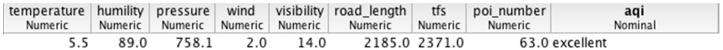
Dataset structure.

**Figure 6:**
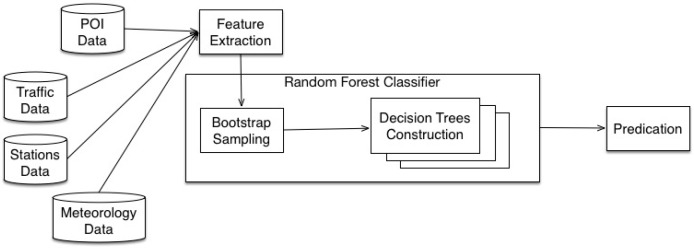
The procedure of RAQ.

In the RAQ algorithm, all data are collected from the urban sensing system including air monitoring station data, meteorology data, traffic data, road information and POI data and necessary features are extracted from heterogeneous data. These features are the most common data in city life. Traffic-related sources like vehicle emissions and POI like factories are the main sources for air pollutants [3]. Meteorology is the main approach for dispersion of air pollutants [3]. These data can represent well the air quality situation. The training dataset includes all the necessary features and is divided into subsets using bootstrap technology. Figure 5 shows the structure of the dataset. A decision tree is constructed on each subset, and the classification is done by aggregating the results generated from all decision trees. Figure 6 shows the procedure of the RAQ algorithm.

### 4.1. Data Collection and Feature Extraction

#### 4.1.1. Meteorology Data

Meteorology data such as temperature and humidity are very important factors that severely affect the concentration and spread of air pollutants. Understanding the behavior of meteorological parameters in the planetary boundary layer is important because the atmosphere is the medium in which air pollutants are transported away from the source, which is governed by the meteorological parameters such as atmospheric wind speed, wind direction, and temperature [32]. In this paper, we use weather monitoring stations as one part of the urban sensing system. Considering the accessibility of the data, we use following meteorology data features: temperature (F_mt_, °C), humidity (F_mh_, %), barometric pressure (F_mp_, mmHg), wind speed (F_mw_, m/s) and visibility (F_mv_, m).

#### 4.1.2. Traffic and Road Data

**Figure 7:**
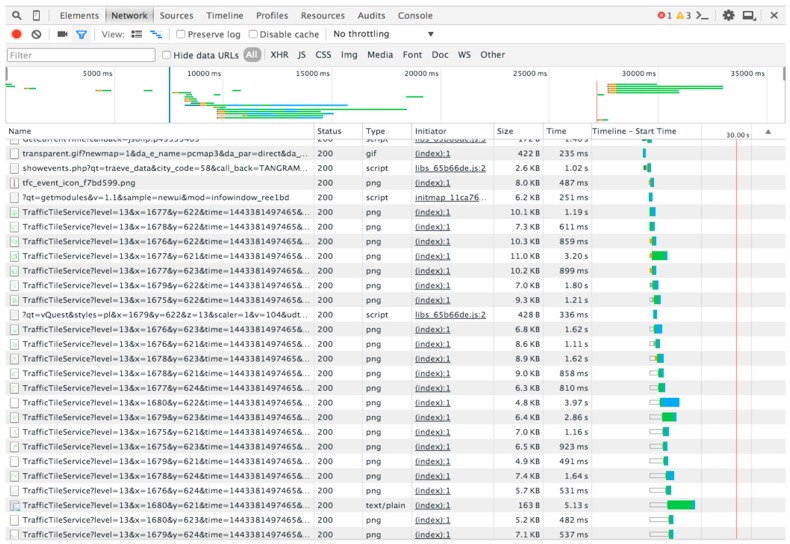
HTTP request analysis by Chrome developer tool.

Traffic is one of the most important factors that affect the air quality. Figure 3 is a sample of the original data that is available from map service providers. In this paper, we rely on two important characteristics of traffic, which are road length (*F_rl_*) and traffic congestion status (*F_tcs_*). If the road is very long and traffic congestion is relatively light, exhaust gas emissions can be at a high level because of the total number of vehicles on this road. Similarly, if a road is short and traffic congestion is heavy. However, we do not have a method or accurate data to quantify these two characteristics directly. Most map service providers offer online maps and real-time traffic status. They do not publish public application interfaces (APIs) for third party developers to access these data, but we can still get some useful hints through analyzing the web http requests of the map. Essentially, these data are collected from GPS equipment installed in cars or speed measurement sensors. These data denote another important part of the urban sensing systems. Figure 7 shows the http request records of a typical Baidu map when we invoke the traffic widget.

As we know, a picture is composed of many pixels, so a picture can be digitized into a matrix. We use the colored pixel distribution to represent the information of road length and congestion status. For each tile grid, we count the quantity of pixels to represent the road. The larger the quantity of pixels, the greater the length of the road is in one tile grid.

**Figure 8:**
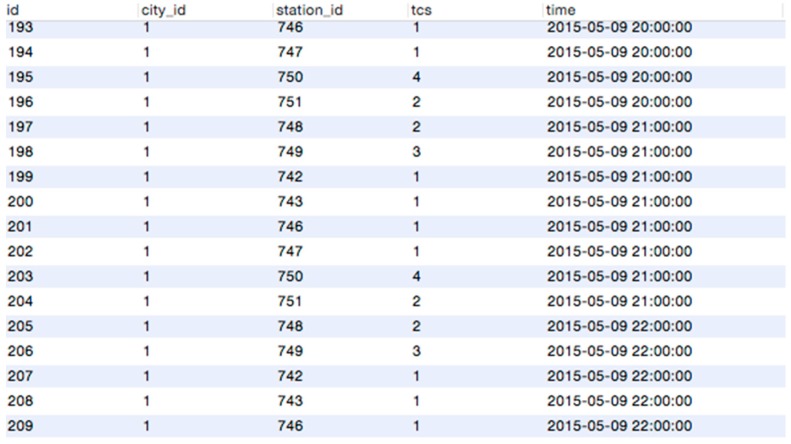
Traffic congestion status.

As shown in Figure 3, traffic congestion status is denoted by different colors (green, orange and red) in the pixels which represent roads. According to the traffic volume of different congestion levels, different weights are assigned to the numbers of pixels in different colors (1, 2 and 5). In Figure 8, the weighted tcs value is calculated by formula *a + 2b + 5c*, where *a* is the number of pixels in green, *b* is the number of pixels in orange and *c* is the number of pixels in red.

#### 4.1.3. POI Data

**Figure 9:**
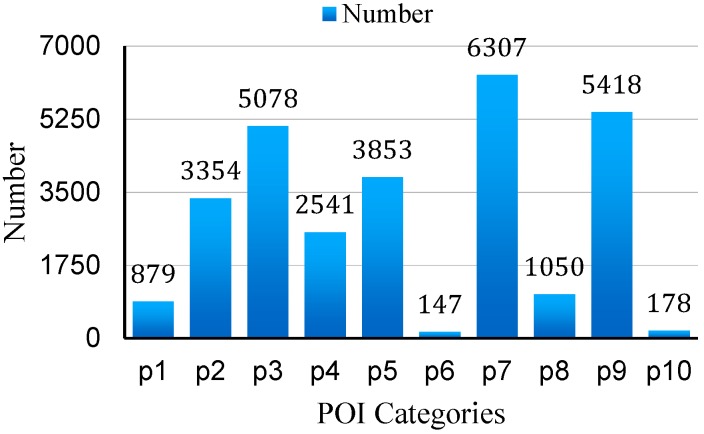
Numbers of POI in Shenyang city.

The category of POIs and their density in a region indicate the land use and the function of the region as well as the traffic patterns in the region, therefore contributing to the air quality inference of the region [24]. For example, shopping streets are more likely to gather more people than parks so there will be more human-related air pollution sources like vehicles. Schools always have more green areas than factories so there are more plants to absorb the air pollutants. Therefore, POI distribution has a strong effect on air quality. These data also imply the significance of human activities in urban sensing systems. In this paper, the number of POI is counted in each tile grid. According to the searching results of Baidu maps and Google Map, the majority of POI are divided into ten categories. Table 2 shows the categories and Figure 9 presents the number of POI (*F_pn_*) in each category.

**Table 2:** POI categories.

Code	POI Category
P1	Transportation
P2	Entertainment
P3	Restaurant
P4	Education
P5	Residential District
P6	Park
P7	Company
P8	Factory
P9	Shopping mall
P10	Gas station

### 4.2. Random Forest Classification

The Random Forest is a general term for ensemble methods using tree-type classifiers $\left\{h(x,\theta_{k}),k=1,...,\right\}$ where the $\left\{\theta_{k}\right\}$ are independent identically distributed random vectors and *x* is an input pattern, $h(x,\theta_{k})$ is a generated classifier [33]. It uses recursive partitioning to generate many trees and then aggregate the results. Each tree is independently constructed using a bootstrap sample of the training data, which subdivides the parameter set first into several parts depending on one of the parameters, and subsequently repeats the process for each part. 

#### 4.2.1. Bootstrap Aggregating (Bagging)

There is usually a single data sample in each class for training. A simple method is to divide the dataset into non-overlapping subsets and construct the trees independently. However, this requires a huge amount of data and it cannot always be guaranteed in different situations. A better way is sampling the original dataset with replacement for a certain times to produce a bootstrap sample. This method ensures that the samples’ distributions are statistically identical with the original data sample [34]. There are *n* records in the original dataset and so the probability of each record is constantly 1/*n*. The probability of not selecting a certain record is (1 – 1/*n*), which results in (1 – 1/*n*)*^n^* when repeated *n* times. 

Assuming the sample size tends to be infinite, the probability can be expressed as lim_n__→∞_(1 – 1/*n*)*^n^* which is equal to *e*^–1^. Therefore, the probability of selecting one record is (1 – *e*^–1^) ≈ ⅔. Thus, in each bootstrap sample there are about ⅔ original samples for training.

#### 4.2.2. Tree Growing and Splitting

As we know, a decision tree starts with one root node. In the following process, the samples are split into different spaces using one of the features including monitoring station data (AQI), meteorology data(MD), traffic(TCS), road information(RI) and POI data.. Therefore, how to select the feature in each split is of great significance for the performance of a decision tree. Information gain [35] is usually used as the criterion for classifiers.

The features selection for each bootstrap sample is randomized. According to bagging theory, random forest is strong classifier based on multiple weak classifiers. Therefore, both the number of data and the number of features of the subset are smaller than original dataset’s. We need T subsets with m features. According to Brieman’s suggestions [33], m is much less than the number of all the features. Brieman suggests three possible values for m: $\frac{1}{2}\sqrt{m},\sqrt{m},\;2\sqrt{m}$. In the evaluation section, we would show four features and 400 subsets are best for our model and dataset.

When splitting the dataset, for each feature candidate, entropy is calculated as in Equation (3):

(3) $$Entropy(c)=-\sum_{i=1}^{k}p(c_{i})\log_{2}p(c_{i})$$

(4) $$p(c_{i})=\frac{N_{i}}{\sum_{i=1}^{k}N_{i}}$$

where *c_i_* is the AQI level *i* which is specified in Table 1, the probability *p*(*c_i_*) is calculated through Equation (2) where *Ni* is the quantity of records in different AQI level and *k* is the number of AQI levels. Therefore, the information gain is defined as shown in Equation (5):

(5) $$Gain(f_{i})=Entropy(c)-\sum_{j=1}^{w}\frac{\left|f_{i}^{j}\right|}{\left|f_{i}\right|}Entropy({f^{j}}_{i})$$

where *f_i_* represents records of the *i_th_* level of tree, *f_i_^j^* are records in *j*th node of the *i*th level of tree, and *w* is the number of nodes in this level.

The process of splitting stops when: (a) the records in one node fall below the threshold value defined by users; (b) the node is pure which means all the records fall into one class. For the terminated node has unordered records, the percentage of different classes are calculated and so the predicted class is defined as in Equation (6):

(6) $$C(i)=Max(p(c_{i}))$$

### 4.3. Prediction

After all the trees are constructed, the unlabeled data are input into all decision trees. For each tree, *p*(*c_i_*) is the estimated probability of the AQI level *i.* The final probability of the AQI level *i* *p’*(*c_i_*) in the random forest is defined in Equation (7), where *T* is the number of decision trees as mentioned before:

(7) $$p^{\prime }(c_{i})=\frac{1}{T}\sum_{k=1}^{T}p(c_{i})$$

The final result is determined by Equation (8):

(8) $$C^{\prime }(i)=Max(p^{\prime }(c_{i}))$$

The pseudocode of RAQ algorithm is described in Algorithm 1.

**Algorithm 1. RAQ**

**Input:**
A dataset *S* with features: *F_mt_, F_mh_, F_mp_, F_mw_, F_mv_, F_ri_, F_tcs_, F_pn_* and labeled AQI level; unlabeled dataset *U*; trees quantity *T*; features quantity *m*;
**Output:**
AQI level1for *T* trees 2randomly select *m* features from *S*;3for *m* features in each node4calculate information gain by Equation (3);5choose maximum gain to split the dataset in the node;6remove used feature from feature candidates;7input unlabeled data into trees;5get predicted AQI level according to Equations (5) and (6);


## 5. Evaluation

### 5.1. Dataset

In the experiments, one-month data from 4 May 2015 to 5 June 2015 is collected and the following four datasets of Shenyang are used which are all available to the public. In our testing period, we use a total of 2701 data to test this algorithm and Shenyang is divided into 1258 grids corresponding to 34 rows and 37 columns. Because all the grids belong to the main city area, all data including meteorology data, traffic data, road information and POI data in these grids are accessible from our data sources. Air quality data is accessible in the areas covered by air monitoring stations.

#### 5.1.1. Monitoring Station Data

The air quality information from the Shenyang monitoring stations includes AQI, the concentrations of CO, NO_2_, SO_2_, O_3_, PM_10_ and PM_25_ and timestamp. Table 3 shows the format of the monitoring station data. Table 4 shows the locations of all the monitoring stations. All the data are collected from the public website [36] whose data are produced by National Department of Environmental Protection. We use the Java programming language to access the API interface hourly and store all the data into a MySQL database.

**Table 3:** Data samples of monitoring stations.

Station_id	Aqi	CO (μg/m^3^)	NO_2_ (μg/m^3^)	SO_2_ (μg/m^3^)	O_3_ (μg/m^3^)	PM_10_ (μg/m^3^)	PM_25_ (μg/m^3^)	Time
747	77	1.802	70	69	63	104	52	2015-05-24 03:00
750	139	2.233	62	70	57	125	106	2015-05-24 03:00
751	82	1.706	73	58	69	100	60	2015-05-24 03:00
741	85	1.942	80	64	43	94	63	2015-05-24 03:00
748	63	1.024	61	62	68	76	37	2015-05-24 04:00
749	67	1.358	60	29	62	81	48	2015-05-24 04:00
742	88	1.646	97	82	12	125	14	2015-05-24 04:00
743	84	0.808	68	167	45	117	52	2015-05-24 04:00
744	98	1.718	66	56	43	92	73	2015-05-24 04:00
745	86	1.333	78	72	9	121	37	2015-05-24 04:00
746	66	1.229	66	24	48	82	45	2015-05-24 04:00
747	63	1.175	58	48	70	75	36	2015-05-24 04:00

**Table 4:** Locations of monitoring stations.

Station_id	Latitude	Longitude
741	41.841445	123.65436
742	41.758166	123.533761
743	41.71694	123.451378
744	41.788094	123.288852
745	41.838551	123.549754
746	41.855605	123.442396
747	41.773208	123.421573
748	41.785295	123.489395
749	41.79609169	123.4084114
750	41.789429	123.373275
751	41.83933982	123.4126515

#### 5.1.2. Meteorological Data

We collect meteorological data including temperature, humidity, barometric pressure, wind speed and visibility from the public website [37]. As Table 5 illustrates, the data format is presented as temperature (*F_mt_*), humidity (*F_mh_*), barometric pressure (*F_mp_*), wind speed (*F_mw_*) and visibility (*F_mv_*).

**Table 5:** Meteorological samples.

Temperature (*F_mt_*, °C)	Barometric Pressure (*F_mp_*, mmHg)	Humidity (*F_mh_*, %)	Wind Speed (*F_mw_*, m/s)	Visibility (*F_mv_*, m)	Time
18.8	748.6	56	2	16.0	2015-05-14 11:00:00
18.3	746.4	50	7	26.0	2015-05-14 08:00:00
17.0	744.6	63	3	12.0	2015-05-14 05:00:00
18.4	743.0	58	1	16.0	2015-05-14 02:00:00
19.7	743.9	63	1	18.0	2015-05-13 23:00:00
18.0	742.6	72	0	7.0	2015-05-13 21:00:00

#### 5.1.3. Road and Traffic Data

There are no public websites that offer statistical road and traffic data. Therefore, we cannot directly get available formatted data. However, most of the map service providers offer online maps and real-time traffic status. They do not publish public API interfaces for third party developers to access these data, but we can still get some useful tips through analyzing the map web http requests. From map services providers [38,39], we collect the traffic map tiles every hour.

#### 5.1.4. POI

**Figure 10:**
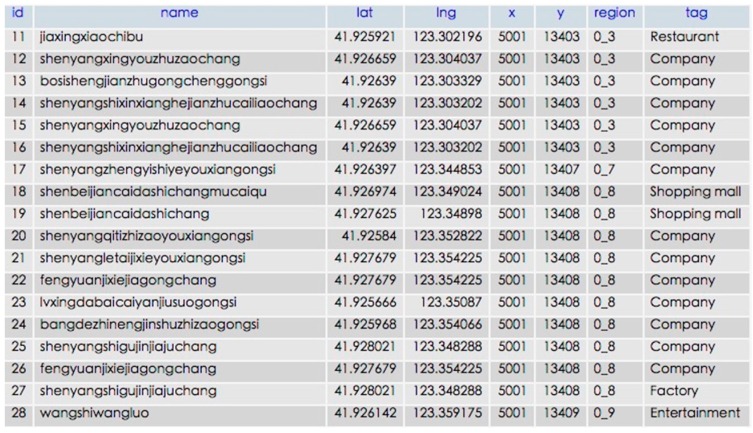
POI Samples in Shenyang.

Thank to Baidu map and Google map service, we can easily get these data from a public interface. Each POI record contains name, latitude, longitude, tag and located tile grids. Figure 10 shows about 28,000 records in the MySQL database.

### 5.2. Evaluation Method

The most accurate criterion for air quality measure is the air quality information from monitoring stations. In this experiment, we use the AQI data from monitoring stations as the reference standard. To construct a random forest, we need to determine two parameters which are the numbers of trees and the number of features used to construct each tree. To choose the best parameters, we use OOB (Out-of-Bag) [33] error to compare RAQ accuracy based on different parameters pairs <#features, #trees> which means the number of features used to construct each tree and the number of trees that are constructed in the random forest. In random forests, the error is estimated internally during the construction of trees. Each tree is constructed using a different bootstrap sample from original data, which about one-third are left out of the bootstrap sample. The one-third sample is used as test cases to be input into the tree and get the classification of each test case. At the end of the run, take the class *j* that got most of the votes every time case *n* was oob [40]. The proportion of times that j is not equal to the true class of *n* averaged over all cases is the oob error estimate [40]. The smaller number of oob, the high accuracy of the model. For the number of features, we increase by one each time from 2 to 8 (total number of features is 8 specified in algorithm ). For the quantity of trees, we increase by 100 from 100 to 1000. Because of the time consumption with more number of trees, we ignore the trees number greater than 1000 and 100 gap is suitable to balance performance and accuracy. To compare this algorithm with others, we use cross-validation method to judge the performance.

### 5.3. Results

#### 5.3.1. Effects of Parameters on Prediction Error Rate

**Figure 11:**
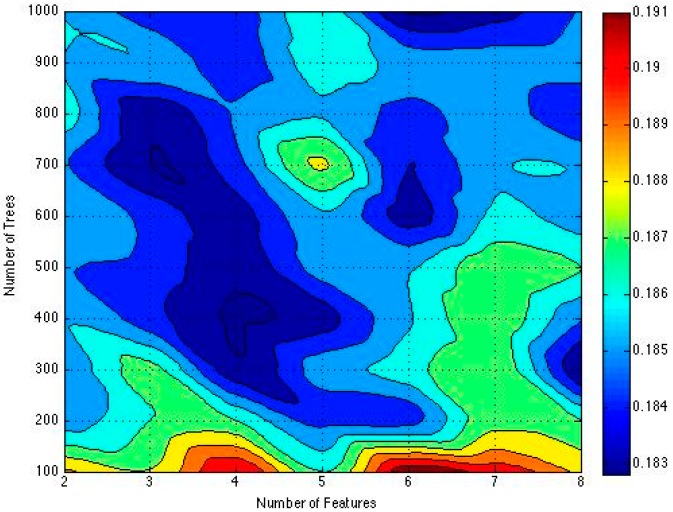
OOB error result distribution.

There are two important factors that affect the performance of a random forest, which are the number of trees and features. Figure 11 shows how the OOB error changes along with the number of features and trees. X-axis is the number of features and Y-axis is the number of trees.

Empirically, for our experiment, we choose integer as the number of features and 100 interval integer as the number of trees, so only the discrete coordinate values such as (2,100), (3,200) are meaningful in this graph. Different colors mean different OOB error values. The deeper the color is, the smaller the oob is. As the graph shows, the OOB errors reach the best when the parameters pairs are <4, 400> and <6, 1000>. Considering less time consumption, we choose <4, 400> as the best parameters pair.

#### 5.3.2. Comparison

For the contrast tests, Naïve Bayes, Logistic Regression, Single Decision Tree and ANN are chosen. Here we use Weka [41] as the tool to conduct all the comparison tests. For Naïve Bayes, there are eight features which are F_mt_, F_mh_, F_mp_, F_mw_, F_mv_, F_ri_, F_tcs_, F_pn_ and six classification categories (C) which are specified in Table 1. In Weka, this algorithm is denoted as weka.classifiers.bayes.NaiveBayesMultinomial. For Logistic Regression, we choose Multinomial Logistic Regression because of the multi AQI levels. In Weka, this algorithm is denoted as weka.classifiers.functions.Logistic. For Single Decision Tree, we choose all the features to construct one single tree for classification. In Weka, this algorithm is denoted as weka.classifiers.trees.REPTree. For ANN, we choose back-propagation neural network with one hidden layer for its simplicity and generality. In Weka, this algorithm is denoted as weka.classifiers.functions.MultilayerPerceptron.

**Figure 12:**
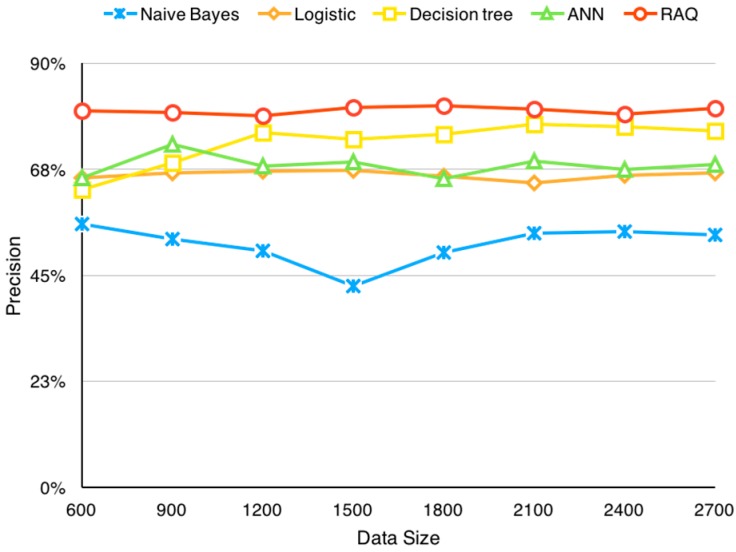
Precision changes according to data size.

After realizing different algorithms, tests are carried out. Table 6 shows the results of the test cases in which Y means correct predictions and N means incorrect predictions. The precision is calculated by the formula Y/(Y + N) where Y is the number of correct predictions and N is the number of incorrect predictions. Figure 12 illustrates how the prediction precision changes as the data size changes. This figure shows RAQ performs steadily, even when the data size is relatively small. Other algorithms are less accurate at all time.

**Table 6:** Precision table of different algorithms.

Algorithm	Precision	Y	N
**NaïveBayes**	52.1%	1408	1293
**Logistic**	66.2%	1790	911
**Decision Tree**	77.4%	2092	609
**ANN**	71.8%	1940	761
**RAQ**	81.5%	2203	498

Besides the precision measurement, we also refer to other measurements including Recall, F-score, Relative Absolute Error (RAE) and Receiver Operating Characteristic (ROC). Recall is the proportion of instances classified as a given class divided by the actual total in that class. F-score is a combined measure for precision and recall calculated as *2∗Precision∗Recall/(Precision + Recall)* where Recall (also known as sensitivity) is the fraction of relevant instances that are retrieved. Relative absolute error is calculated by the following formula:

$$RAE=\frac{\sum_{i=1}^{N}\left|\theta_{i}-r_{i}\right|}{\sum_{i=1}^{N}\left|\overset{-}{\theta }-r_{i}\right|}$$

where $\theta_{i}$ is the estimated value, $r_{i}$ is the real value, $\bar{\theta }$ is the average value, N is the number of test cases. ROC shows how the number of correctly classified positive examples varies with the number of incorrectly classified negative examples [42].

**Table 7:** Indexes of different algorithms.

Algorithm	Recall	F-Score	ROC	RAE
**Naive Bayes**	0.521	0.529	0.7	84.9%
**Logistic**	0.663	0.649	0.785	75.8%
**Decision Tree**	0.775	0.769	0.888	47.4%
**ANN**	0.718	0.707	0.829	60.9%
**RAQ**	0.816	0.814	0.928	36.9%

**Figure 13:**
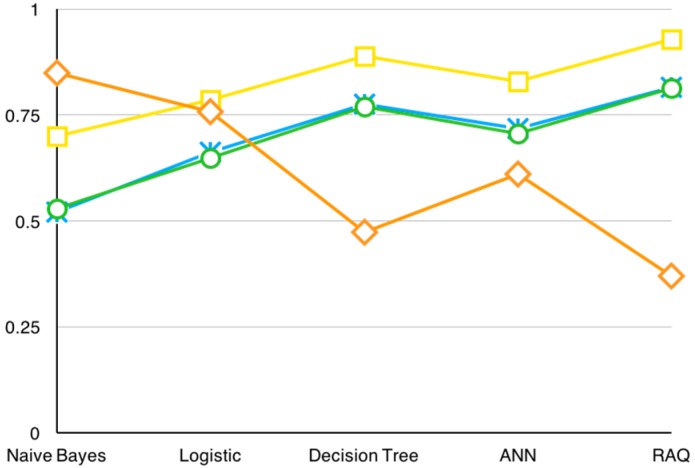
Indexes chart of different algorithms.

Based on our dataset, these measurements also show that RAQ performs better than others in this specific problem. Table 7 shows the original data of the experiments result and Figure 13 illustrates these data in chart form.

## 6. Conclusions

In this paper, with the public data in the urban sensing system, our model predicts the AQI of all the regions in Shenyang based on the AQI published by 11 air quality monitoring stations, meteorology data reported by weather stations, road information and real-time traffic status collected from Baidu Map and Google Maps and the POI distributions provided by Baidu Map and Google Maps. We use a random forest algorithm to predict all the uncovered regions in the downtown area. In Shenyang, this algorithm finally results in an overall precision of 81% for AQI prediction. This experimental result outperforms that of Naïve Bayes, Logistic Regression, single decision tree and ANN. All of these data are directly or indirectly available on the Internet. This shows that the algorithm could be easily applied for other cities. RAQ makes use of historical data for model training but ignores the real-time data. Our work will be extended to support online learning so daily data can be used to improve the performance of the air prediction algorithm.
